# Analysis of the Clinical Characteristics and Pituitary Function of Patients in Central China With Rathke’s Cleft Cysts

**DOI:** 10.3389/fendo.2022.800135

**Published:** 2022-02-28

**Authors:** Lixia Zhang, Xueyuan Li, Chong Li, Zhifang Wang, Lili Zheng, Guijun Qin, Shoujun Wang, Lijun Xu

**Affiliations:** Department of Endocrinology, The First Affiliated Hospital of Zhengzhou University, Zhengzhou, China

**Keywords:** Rathke’s cleft cyst, hypopituitarism, pituitary tumor, central precocious puberty (CPP), central of China

## Abstract

**Objective:**

A Rathke’s cleft cyst (RCC) is a common, benign, cystic disease that often leads to hypophyseal dysfunction or head symptoms. The relationship between RCCs and pituitary gland function is not clear. We therefore carried out a study to examine this relationship in greater detail.

**Methods:**

The study was a retrospective, cohort design in patients diagnosed with a RCC between January 2019 to July 2021 at the First Affiliated Hospital of Zhengzhou University, China.

**Results:**

A total of 221 patients were enrolled and then divided into study cohorts according to the diameter of the RCC, clinical manifestations, and surgical treatment received. The majority of patients were symptomatic (143/221), including 83 cases of dizziness and headache, 9 of vision loss and visual field defect, and 2 of diabetes insipidus. 52 cases had abnormal pituitary function, with 8 cases interestingly showing high adrenocorticotropic-hormone (ACTH) and cortisone levels, while 8 juvenile cases had developed central precocious puberty. Patients with larger RCCs were more likely to present with headaches and dizziness, with subjects who suffered from these symptoms having high ACTH and cortisone levels.

**Conclusion:**

Although the size of a RCC is not an important factor influencing hypopituitary function, we consider that endocrine evaluation should be carried out in all patients with a RCC.

## Introduction

A Rathke’s cleft cyst (RCC) is a common, benign, cystic lesion that originates from the remnant tissue of a Rathke’s cyst (1). Different autopsy studies have shown the prevalence of RCCs ranges from 13% to 22%. The cysts are often located in the saddle or upon the saddle. Most patients have no clinical manifestations, with symptomatic Rathke’s cysts being relatively uncommon (2). Medical management of RCCs has not yet been standardized (3), although it is generally accepted that clinical and radiological monitoring is sufficient for cases of incidental asymptomatic RCCs. However, it is possible that RCC-related pituitary dysfunction and other manifestations, such as headaches and visual disturbances may be underestimated by this approach (4). Because of the benign features of RCC, there have only been a small number of clinical studies on pituitary function in patients with RCC in China, with pituitary dysfunction often ignored in diagnosis and treatment. To further assess pituitary function in patients with RCC, we performed a cross-sectional, retrospective study of patients with RCCs, diagnosed by magnetic resonance imaging (MRI) or histology following surgery.

## Patients And Methods

### Patients

According to the guidelines of the ZhengZhou University Medical Ethical Committee, the data of patients diagnosed with a RCC who were admitted to the First Affiliated Hospital of Zhengzhou University from January 2019 to July 2021 were collected to establish a retrospective observational cohort study. Study eligibility criteria included a radiologically defined RCC and assessment of pituitary function. A total of 221 patients were enrolled in the study including 22 patients who underwent surgery and had a pathological diagnosis and 199 patients with a presumed diagnosis following an MRI. Basic information of the patient’s gender, age, chief complaint, symptoms and signs were collected. Data on the function of endocrine glands were collected, including thyroid function free serum thyroxine (FT4), free triiodothyronine (FT3), thyroid stimulating hormone (TSH), serum ACTH and cortisone (at 8am and 0am), serum gonadotropin [luteinizing hormone (LH) and follicle-stimulating hormone (FSH)], prolactin (PRL), growth hormone (GH) and insulin-like growth factor-1 (IGF-1). Information on MRI imaging suprasellar area and pathological diagnosis was also recorded.

### Data Analysis

RCC-related clinical manifestations were determined according to the patient’s medical history and included headache, dizziness, decreased vision, visual field defects, sexual dysfunction, and diabetes insipidus. Pituitary dysfunction was diagnosed based on laboratory results and clinical signs and symptoms (The diagnostic criteria are detailed in **Appendix 1**) and defined as either hypoadrenalism(secondary), hypothyroidism(secondary), growth hormone deficiency, hypogonadotropic hypogonadism, precocious puberty, delayed puberty, or asymptomatic. For analysis, the data was grouped according to clinical manifestations, pituitary function, treatment methods, and the diameter of the RCC.

### Statistical Analysis

SPSS16 software was used for the statistical analyses. Numeric variables were expressed as mean ± standard deviation (SD) and qualitative variables as numbers and percentages (%). The non-parametric Kruskal Wallis test or the Pearson 2 test were used to compare categorical variables. *P* values < 0.05 were considered statistically significant.

## Results

### General Characteristics

A total of 221 patients with a mean age of 40.31 ± 18.58 years (range, 5 - 82 years), were enrolled in the study. Of the 221 patients, 117 (52.94%) were aged 30-50 years and 32 were younger than 18 years old. There were 107 males (aged 41.01 ± 19.79 years) and 114 females (aged 39.63 ± 17.42 years) in the study cohort. There was no significant difference in age between the male and female patients (t=0.561, *p*=0.575). 22 cases underwent surgery and were diagnosed pathologically, while the remaining 199 cases who received conservative treatment were diagnosed by a MRI of the sellar area. The ages of patients who accepted surgery or conservative treatment were 37.59 ± 19.29 years and 40.61 ± 18.52 years, respectively. The difference in age between the two groups was not statistically significant. A similar number of males and females underwent surgery (n=11 for each gender).

### Clinical Manifestations

143 patients were symptomatic, including 83 cases who suffered from dizziness and headache, 9 cases who suffered vision loss or visual field defection, and 2 patients with diabetes insipidus. 52 patients showed symptoms due to abnormal pituitary function including afraid of the cold, poor appetite, loss of libido or gonadal growth retardation, early puberty development, and slow height growth. The remaining 78 cases were asymptomatic. When grouped according to clinical symptoms, the patients with headache and dizziness had larger cysts, with a mean diameter of 15.05 ± 5.92 mm, whereas patients without headache or dizziness had significant smaller cysts with a mean diameter of 0.30 ± 6.98 mm (*p* = 0.001).

### Evaluation and Analysis of Endocrine Function

A total of 52 patients had hypopituitarism, including 23 cases of central hypothyroidism, 10 of hypogonadotropic hypogonadism, 5 of hypoadrenalism, 21 of growth hormone deficiency (14 partial deficiency and 7 complete deficiency), and 8 of increased ACTH level. As shown in **Table 1**, serum cortisone levels at 8am were highest in patients without hypopituitarism, while there was no significant difference in either the size of the cyst or age of the patient between cases with or without hypopituitarism.

**Table 1:** Comparison of endocrine function indices between patients with or without hypopituitarism.

	Hypopituitarism n=52	Without Hypopituitarism n=169	*P*
Age (yr)	39.48 ± 20.1	40.57 ± 18.14	0.713
Diameter of RCC (mm)	12.41 ± 8.18	13.19 ± 6.25	0.620
FT3 (pmol/L)	4.96 ± 2.66	4.91 ± 1.33	0.843
FT4 (pmol/L)	11.32 ± 8.63	12.14 ± 4.33	0.363
TSH (μiu/mL)	2.2 ± 1.64	3.33 ± 11.88	0.496
IGF-1 (ng/mL)	181.54 ± 95.66	194.74 ± 96.14	0.514
GH (ng/mL)	0.68 ± 1.74	1.26 ± 4.57	0.497
FSH (mIU/mL)	9.47 ± 15.75	12.16 ± 17.09	0.254
LH (mIU/mL)	5.64 ± 7.85	9.11 ± 10.74	0.045*
PRL (ng/mL)	25.58 ± 24.52	25.09 ± 30.52	0.922
ACTH 0am (pg/mL)	9.36 ± 9.07	11.96 ± 12.79	0.270
ACTH 8am (pg/mL)	20.45 ± 17.39	42.87 ± 83.92	0.075
Cortisone 0am (μg/dL)	3.97 ± 5.43	3.59 ± 7.37	0.778
Cortisone 8am (μg/dL)	7.33 ± 5.96	12.12 ± 7.93	0.000*
24h UFC (nmol/d)	231.79 ± 230.22	290.78 ± 111.05	0.116

24h UFC, 24-hour urine free cortisol, *P < 0.05.

Function of hypothalamic-pituitary target gland axes, as shown above, only serum cortisone level at 8am was higher in patients without hypopituitarism, there was no significant difference in either the size of the cyst or age of the patient between cases with or without hypopituitarism. Levels of FT3, FT4, TSH, IGF-1, GH, FSH, LH, ACTH, cortisone at 0am, and 24hUFC in patients with hypopituitarism were slightly lower than that of patients without hypopituitarism, but not statistically significant.

### Imaging Examination

**Figure 1:**
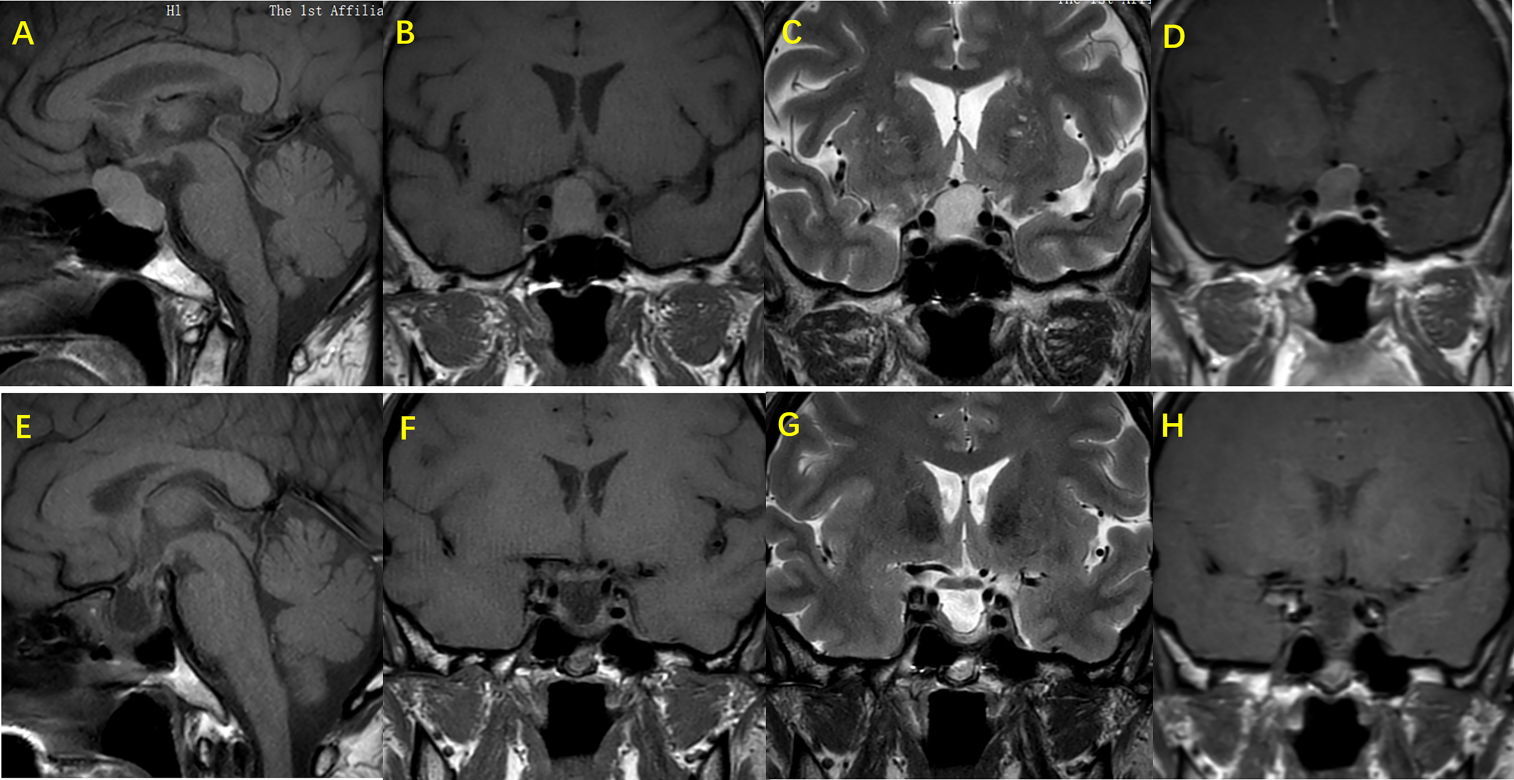
Images of MRI and gadolinium-enhanced MRI sequencing for sella region in RCC patient. Female patient, age 47, suffers headache as the main symptoms, MRI shows a snowman appearance of the RCC, the cyst present isointense on T1W imaging. **(A, B)** and hypertense on T2W imaging. **(C)** and the wall has been partialy enhanced with gadolinium administration **(D)** In the preoperative picture of the patient, the RCC is compressing the sella - supraselar region causing compression of optic chiasm, while pituitary stalk is not visible as the result of compression. Pictures **(E–H)** show the 6 months post - operation evaluation of the pituitary gland. Hypertense on T1W **(E, F)** and heterogeneous intensity on T2W imaging **(G)** present in the sela - suprasellar region, with irregular enhancement after gadolinium administration. Also, pituitary stalk appears although it has been shifted, and the compression of optic chiasm is seen relieved.

All patients underwent a pituitary MRI examination, with the presumptive diagnosis of RCC made by at least two doctors (**Figure 1**). In 154 cases, the largest diameter of the cysts was <10 mm (mean 5.50 ± 2.42 mm), and in 67 cases the diameter of the cysts was ≥10 mm (mean 16.35 ± 5.20 mm). The patients were divided into two groups according to the diameter of the cysts. Those with a largest diameter <10 mm were defined as group A, and patients with a largest diameter of ≥10 mm as group B. Comparison of these two groups showed that the difference in cyst diameter was statistically significantly, whereas the gender proportion and age at onset was similar. As shown in **Table 2**, the incidence of headache and dizziness was higher in group B. Among the pituitary function indexes, only the ACTH level at 8 am was significantly different in the two groups, but interesting, the level of ACTH in group B was higher than that in group A (**Table 3**).

**Table 2:** Comparison of general information between groups A and B.

Name	Diameter of RCC		Total	*P* value
	<10mm (Group A)	≥10mm (Group B)		
With dizziness or headache	36	47	83	
Without dizziness or headache	118	20	138	0*
Male	81	26	107	
Female	73	41	114	0.059
<18years	24	8	32	
Adult	130	59	189	0.479

*P < 0.05.

The incidence of headache and dizziness was higher in patients with large RCC. There is no difference in gender and age distribution between the two groups.

**Table 3:** Comparison of endocrine function between groups A and B.

	Diameter <10mm	Diameter ≥10mm	*P*
Diameter of RCC (mm)	5.50 ± 2.42	16.35 ± 5.20	0.000*
Age (yr)	40.13 ± 19.11	40.169 ± 17.13	0.988
FT3 (pmol/L)	5.05 ± 1.51	5.12 ± 2.73	0.891
FT4 (pmol/L)	12.68 ± 5.98	12.18 ± 8.75	0.775
TSH (μiu/mL)	4.85 ± 15.18	3.65 ± 15.91	0.724
IGF1 (ng/mL)	223.69 ± 131.20	174.33 ± 75.05	0.115
GH (ng/mL)	0.94 ± 2.28	0.62 ± 0.86	0.451
FSH (mIU/mL)	11.76 ± 17.95	10.44 ± 15.37	0.142
LH (mIU/mL)	7.73 ± 8.82	8.61 ± 10.04	0.694
PRL (ng/mL)	21.22 ± 1346	23.64 ± 26.26	0.669
ACTH 0 am (pg/mL)	10.72 ± 10.37	14.85 ± 17.7	0.319
ACTH 8 am (pg/mL)	24.77 ± 15.48	37.33 ± 33.95	0.022*
Cortisone 0 am (μg/dL)	4.12 ± 5.57	3.62 ± 4.52	0.721
Cortisone 8 am (μg/dL)	10.18 ± 5.64	10.48 ± 6.26	0.841

*P < 0.05.

Among the pituitary function indexes, only the ACTH level at 8 am was significantly different in the two groups, but interesting, the level of ACTH in group B was higher than that in group A.

### The Relationship Between Endocrine Function and Clinical Manifestations

Headache and dizziness were the most common symptoms observed in our study cohort. We analyzed the difference between patients with head symptoms (named as group C)and those without head symptoms(named as group D). There was no significant difference in gender distribution between groups C and D, although the size of the cysts was significantly different (15.05 ± 5.92 mm vs. 10.30 ± 6.98 mm, respectively, *P*<0.05). The levels of serum ACTH at 0 am was significantly higher in patients with headache and dizziness than in patients without these symptoms (15.24 ± 17.29 vs. 9.16 ± 7.17 pg/mL, respectively, *P*<0.05). Eight patients had increased serum ACTH and cortisone levels, with all these patients suffering from headache or dizziness. The incidence of these symptoms was significantly different compared with those in patients with normal ACHT and cortisone levels (8/8 vs. 75/213, *P <*0.001). Patients with increased or normal serum ACTH and cortisone levels also had larger RCCs than patients without headache and dizziness (as described above). Compared with age-matched children with normal stature, eight juveniles with RCC were short in stature, all of whom received growth hormone excitation tests. The basic level of serum GH showed no difference between these two groups (0.41 ± 0.44 vs. 2.76 ± 3.80 ng/mL, *P*>0.05), whereas the peak level of GH was lower in short children compared to children with a normal stature (5.38 ± 2.28 vs. 10.04 ± 1.59 ng/mL, *P*<0.05). The serum levels of IGF-1 (228.80 ± 63.22 vs.358.72 ± 187.92 ng/mL) and LH (1.70 ± 1.35 vs. 4.97± 3.49) were also lower in short juveniles compared to that in juveniles with normal height (both *P*<0.05). However, there was no difference in cyst diameter between these two groups (7.00 ± 7.71 vs. 12.83 ± 5.076 mm, respectively, *P*>0.05).

Eight juveniles in the study (2 males, 6 females, mean age 7.88 ± 2.17 years) were diagnosed with precocious puberty, with a mean diameter of RCC of 9.80 ± 1.69 mm. With the exception of a pituitary-gonadal axis disorder, no other pituitary-target organ axis was affected. Unfortunately, no age-matched children were enrolled in the study as a control group.

### Treatment Methods

**Figure 2:**
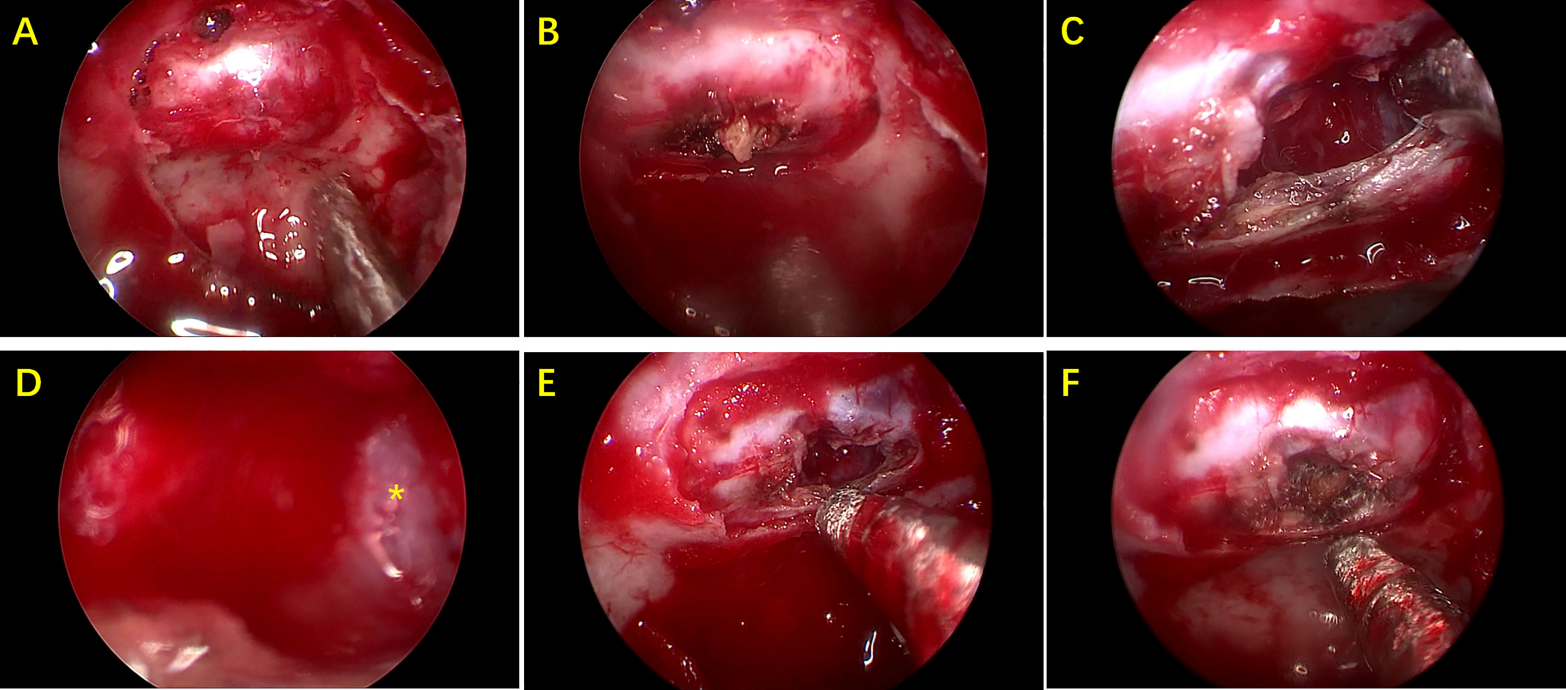
Pictures during endoscopic endonasal transsphenoidal surgery. **(A)** Exposure of sellar dura mater. **(B)** After the dura mater is cut open, there is some cheese-like substance. **(C)** Capsule contents are cleared up. **(D)** Local enlarged view of capsule wall(*). **(E)** Final status of cyst wall fistula. **(F)** A small amount of hemostatic material are filled in the capsule.

**Figure 3:**
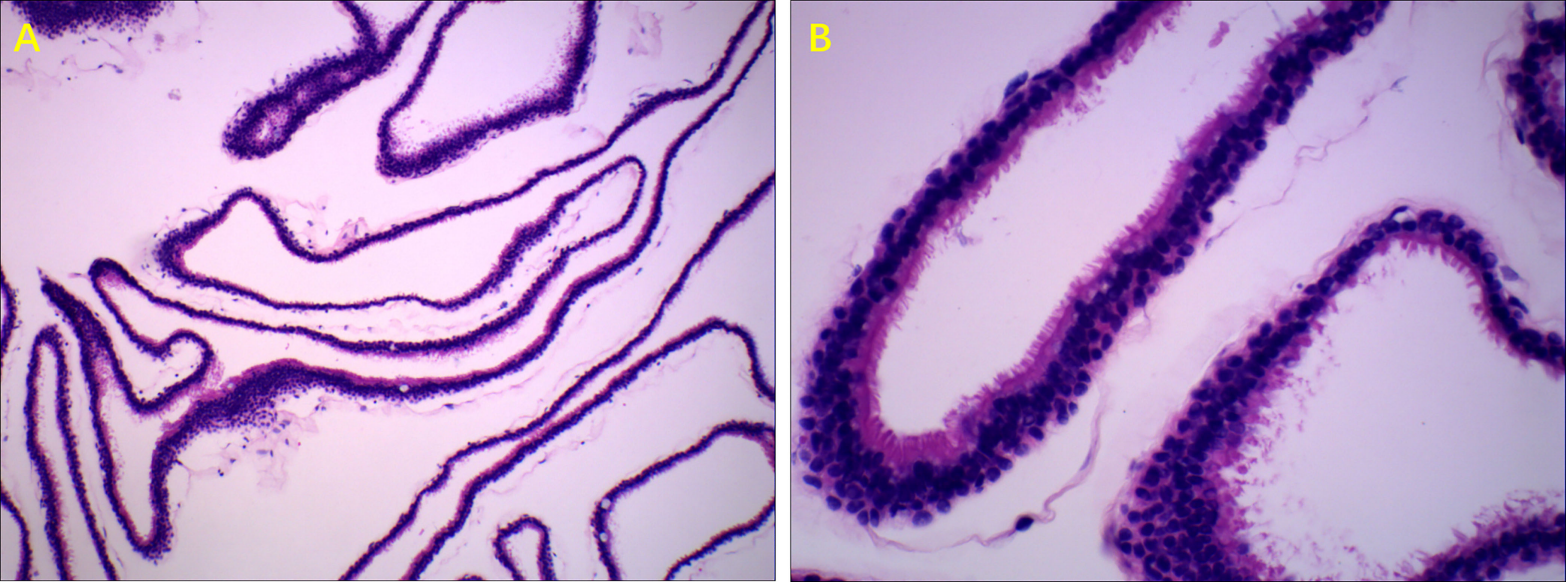
Typical pathologic manifestations of RCC. Ciliated columnar epithelium of RCC, H&E stain. **(A)** (100× magnification). **(B)** (400× magnification).

A total of 22 patients received surgery for RCC, most of who were symptomatic patients, including those with headaches, dizziness, decreased vision, and visual field defects. All the patients underwent endoscopic endonasal transsphenoidal dissection of RCC surgery. A round fistula of no less than 5mm in diameter was produced in the wall of each RCC (**Figure 2**). The contents of the RCC were described as a jelly-like substance of different colors. After clear-up of the RCC-contents, the cystic parietal cells were destroyed to the great extent with a scraping circle to reduce the recurrence rate of RCC. A small amount of hemostatic material was filled in the operation cavity. Clinical pathological reports described a powder-stained amorphous substance, with the wall of the capsule composed of a single layer of cubic epithelial ciliated columnar epithelial cells or cubic epithelial cells (**Figure 3**). The maximum diameter of the cysts in the patients who received surgery was significantly greater than that of patients who received conservative treatment (17.70 ± 5.57 mm vs. 11.92 ± 6.58 mm, respectively, *p*<0.05). The level of ACTH at 0 am was higher in patients who underwent surgery than that measured in patients who received conservative treatment (30.76 ± 28.57 vs. 9.74 ± 8.046 pg/mL, respectively, *p*<0.05). The other indicators of endocrine function were similar in the two treatment groups.

## Discussion

We conducted a retrospective observational study to analyze the clinical manifestations of RCCs and the effect of a RCC on pituitary function. The study carried out in central China showed that RCC occurs at different ages and predominantly in females. Most patients with RCC are symptomatic and have head symptoms and abnormal pituitary function, not only hypopituitary, but also hyperfunction, such as high ACTH levels and precocious puberty. In the current study, the size of the RCC was not related closely to hypopituitary. The study also provided suggestions regarding evaluation of endocrine function in patients with RCC. A study conducted by Mizue Fujii on the Japanese also came up with similar results, RCC, even small ones, can cause pituitary dysfunction (5).

The findings of the study on the general characteristics of the patients were consistent with those of previous reports. The age distribution ranged from children to elderly people, with the highest incidence of RCCs in people aged 30-50 years. The majority of patients were symptomatic, with headache being the most common symptom (6), with an incidence of 64.7% in this study, similar to that reported in other studies (7, 8). We observed that about one third (67/221) of patients had a large RCC, and as expected the size of the RCC was related to the presence of headache and dizziness. Our data also showed that the incidence of headache and dizziness was higher in patients with a RCC diameter >10mm, a finding consistent with those reported in previous literature (9, 10).

Among the pituitary function indices, the level of ACTH at 8 am was significantly different between patients with different sized RCCs, and it is interesting that the level of ACTH was highest in patients with a larger RCC. We consider that headache and dizziness caused by a large RCC may be the reason for this increase in ACTH level (11), pain is a stressful stimulus that is likely to elicit ACTH and cortisol secretion and is commonly associated with hypercortisolism, ACTH-cortisone is a key player in the stress response (12). This is consistent with result in this study, the incidence of headache and dizziness was higher in patients with large RCC, and all the patients with a high ACTH level suffered from headache or dizziness. Other reasons for these results should be considered, because patients who underwent surgery received a pathological diagnosis, while in other symptomatic patients who received conservative treatment, the presence of a RCC and ACTH secreting pituitary microadenoma (13) or even a pituitary adenoma and concomitant RCC (14–16) could not be definitely excluded. Although both these states have been reported in the literature, we showed that none of our patients had been diagnosed with Cushing syndrome, while no symptoms and signs related to this syndrome were identified in the medical records.

Compared to other research, our study showed a low prevalence of hypopituitarism (17, 18), especially hypogonadotropic hypogonadism, with only 10 patients complaining about this symptom. However, we observed lower LH levels in patients with hypopituitarism. Consistent with the culture of central China, people are ashamed to talk about sexual function and this hesitation may be responsible for the low incidence of hypopituitarism we observed. Of note, no correlation between hypopituitarism and RCC size was observed in our study, a finding similar to that reported by other literature (6). However, a significant positive relationship between RCC size and the number of impaired hormones has been reported previously, although there is no consensus regarding this association.

RCC may be one of the causes of central precocious puberty, although this relationship is also controversial (7, 19, 20). In our study, eight cases showed precocious puberty, mainly girls, a finding different from data reported in some other papers (7). Han Hyuk Lim et al. reported smaller RCCs in cases of precocious puberty, although in our study the diameter of RCCs in patients with or without precocious puberty was similar. The mechanism of how RCCs leads to precocious puberty is unclear. Possible mechanisms reported as following, larger cysts may cause extreme pressure on the pituitary, lead to precocious puberty by interrupting a neuronal inhibitory mechanism for pituitary gonadotropin release. While smaller cysts may elicit endocrine dysfunction through inflammation of the pituitary or by interruption of normal inhibitory pathway. In our study there were also eight cases of RCC in people with a short stature who had low levels of GH, IGF-1, and LH and were diagnosed with delayed puberty or growth hormone deficiency, both of which may result from a RCC (21–23). The gene mutation is consider to be responsible role, such as the transcription factor ISL1, It’s expressed in pituitary gland stem cells and thyrotrope and gonadotropin lineages, with the conditional Isl1 deletion causing development of multiple Rathke’s cleft-like cysts, with 100% penetrance (24, 25).

Surgery and conservative treatment were both used for treatment of RCC in the patients in our study. Symptoms and signs were the basis for these treatment plans, and although our results showed that most patients were symptomatic, fewer patients underwent surgery compared with that reported in other literature (26). Chinese conservative thinking considers that surgery will destroy vitality, especially surgery on the head.

The pathogenesis of RCC remains controversial, classic RCCs are lined by a single layer of cuboidal or ciliated columnar epithelium, while stratified squamous epithelial cells lining a portion of RCCs are reported. This finding favors the hypothesis that RCCs and craniopharyngiomas, which are lined with multi-stratified squamous epithelium, may represent two extremes of a continuum of cystic sellar lesions. Due to the high rate of relapse and progressions (17, 24, 27, 28) and the homologous etiology of RCCs and craniopharyngioma (24, 29), patients with a RCC should receive regular clinical follow-up, especially juvenile patients.

## Limitations

Our study had some limitations, the first of which was its retrospective cross-sectional design. Second, patients enrolled in the study were treated at a single medical center. Third, follow-up studies are needed to better understand the long-term effects of management of RCCs.

## Conclusion

This study analyzed the impact of RCC on pituitary function and its relationship with RCC-related clinical manifestations. The majority of patients with RCC were symptomatic, mainly suffering from headaches and dizziness. RCC should therefore be considered as a possible reason for these symptoms. We observed that hypopituitary function was not closely related to cyst size, suggesting that clinicians need to evaluate endocrine function in patients with an identified RCC.

## Data Availability Statement

The original contributions presented in the study are included in the article/supplementary material. Further inquiries can be directed to the corresponding author.

## Ethics Statement

The studies involving human participants were reviewed and approved by Ethics Committee of Zhengzhou University, Zhengzhou University. Written informed consent to participate in this study was provided by the participants’ legal guardian/next of kin.

## Author Contributions

LXZ and XL performed the data analyses and wrote the manuscript. XL contributed to the conception of the study. CL and ZW performed the collection of data collection and recording. LLZ, GQ, and SW helped perform the analysis with constructive discussions. LX contributed significantly to analysis and manuscript preparation. All authors contributed to the article and approved the submitted version.

## Conflict of Interest

The authors declare that the research was conducted in the absence of any commercial or financial relationships that could be construed as a potential conflict of interest.

## Publisher’s Note

All claims expressed in this article are solely those of the authors and do not necessarily represent those of their affiliated organizations, or those of the publisher, the editors and the reviewers. Any product that may be evaluated in this article, or claim that may be made by its manufacturer, is not guaranteed or endorsed by the publisher.

## Diagnostic Criteria

1. Secondary Hypoadrenalism, defined as patients with low level of cortisone in serum or urine (serum cortisone < 5μg/dl, 24hUFC < 73nmol/d Chemiluminescence immunoassay), and low serum ACTH at 8am (serum ACTH < 7pg/ml, Chemiluminescence analysis)
2. Secondary Hypothyroidism, defined as patients with low level of serum FT4 or FT3 (FT4 <7.9 pmol/ml, or FT4 <3.28 pmol/ml, Chemiluminescence immunoassay), and low TSH in serum (TSH < 5.91U μ iu/ml, Chemiluminescence immunoassay)
3. Growth hormone deficiency (GHD), GHD is defined by a peak GH response to insulin-induced hypoglycemia (insulin tolerance test, ITT), less than 5 ng/ml or 10 ng/ml, named complete growth hormone deficiency and partial growth hormone deficiency respectively.
4. Hypogonadotropic hypogonadism (HH). In teenagers (<18 years), HH diagnosed by low blood testosterone/ estrogen levels and low pituitary hormone level (LH), confirmed by GnRHa provocation test, peak LH response to gonadotropin stimulation test (0.1mg im) less than 12 mIU/ml (Chemiluminescence immunoassay). In adults with RCC, HH diagnosed by low blood testosterone/ estrogen levels and low pituitary hormone levels.
5. Abnormal puberty, include precious puberty and delayed puberty. precious puberty refers to puberty begins before age 8 in girls and before age 9 in boys, delayed puberty is defined by the absence of testicular development in boys beyond 14 years (or a testicular volume lower than 4 ml) and by the absence of breast development in girls beyond 13 years.
